# Human Endometrial Pericytes: A Comprehensive Overview of Their Physiological Functions and Implications in Uterine Disorders

**DOI:** 10.3390/cells13171510

**Published:** 2024-09-09

**Authors:** Yiqun Tang, Caroline Frisendahl, Terhi T. Piltonen, Riikka K. Arffman, Parameswaran Grace Lalitkumar, Kristina Gemzell-Danielsson

**Affiliations:** 1 WHO Collaborating Centre, Division of Neonatology, Obstetrics, Gynecology, and Reproductive Health, Department of Women’s and Children’s Health, Karolinska University Hospital, Karolinska Institutet, SE 17176 Stockholm, Sweden; yiqun.tang@ki.se (Y.T.); caroline.frisendahl@ki.se (C.F.); lalit.kumar@ki.se (P.G.L.); 2 Department of Obstetrics and Gynecology, Research Unit of Clinical Medicine, Medical Research Centre, Oulu University Hospital, University of Oulu, 90220 Oulu, Finland; terhi.piltonen@oulu.fi (T.T.P.); riikka.arffman@oulu.fi (R.K.A.)

**Keywords:** pericyte, mesenchymal stem cell, endometrial repair, regeneration, endometrial fibrosis, uterine disorder

## Abstract

Pericytes are versatile cells integral to the blood vessel walls of the microcirculation, where they exhibit specific stem cell traits. They are essential in modulating blood flow, ensuring vascular permeability, and maintaining homeostasis and are involved in the tissue repair process. The human endometrium is a unique and complex tissue that serves as a natural scar-free healing model with its cyclical repair and regeneration process every month. The regulation of pericytes has gained increasing attention due to their involvement in various physiological and pathological processes. However, endometrial pericytes are less well studied compared to the pericytes in other organs. This review aims to provide a comprehensive overview of endometrial pericytes, with a focus on elucidating their physiological function and potential implications in uterine disorders.

## 1. Introduction

Pericytes, also known as Rouget cells, were first identified and named by Zimmerman in 1923 [1]. In the early twentieth century, a population of contractile cells was discovered enveloping the wall of the blood vessels [2]. They were given the name “pericytes” owing to their anatomical position in a perivascular location, surrounding the arterioles and capillaries, close to endothelial cells [3]. Pericytes are highly branched interstitial cells of mesenchymal origin found across various organs and tissues [4]. They have critical roles in regulating blood flow, mediating vascular permeability, maintaining tissue homeostasis, and facilitating tissue repair and regeneration.

The human endometrium, a unique tissue rich in microvasculature, undergoes approximately 400 cycles of proliferation, differentiation, breakdown, shedding, and repair throughout a woman’s reproductive lifespan [5]. Endometrial growth and differentiation are tightly regulated by steroid hormones during the proliferative and secretory phases of the menstrual cycle [6]. The absence of pregnancy leads to a drastic decline in progesterone levels, resulting in vasoconstriction of the basal arteries and causing the functional layer of the endometrium to disintegrate and shed during menstruation. This process typically occurs swiftly without scarring, along with repair and re-epithelialization being completed within 48 h of the onset of shedding [7]. The initial repair phase of endometrial regeneration is independent of steroid hormones [8], involving a complex interplay of inflammatory, coagulation, and angiogenic factors needed for tissue stabilization and homeostasis during the early proliferative phase [9]. Endometrial vascular remodeling plays a crucial role in minimizing blood loss during menstruation.

However, the cellular and molecular mechanisms of endometrial vascular remodeling and the regeneration of various cell lineages, particularly the involvement of pericytes, remain poorly understood and warrant further investigation. This review aims to offer a comprehensive view of the origin, molecular markers, characteristics, and functions of endometrial pericytes, as well as their implications for uterine disorders. Elucidating the role of pericytes in endometrial physiology and pathology holds promise for developing pericyte-targeted therapies for uterine disorders.

## 2. Characterization of Pericytes in the Endometrium

### 2.1. Origin

**Figure 1:**
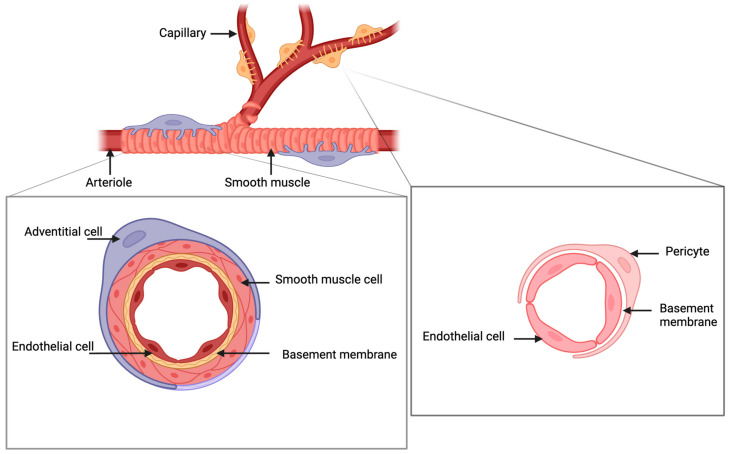
Schematic illustration of perivascular cells in the endometrium. Created using BioRender.

Pericytes are notable for their location along the arterioles and capillaries, where they form close associations with endothelial cells [10]. Blood vessels consisting of pericytes and endothelial cells are some of the first organs to develop from the embryonic mesoderm during embryonic development [11]. In mature tissues, pericytes are situated along the outer wall of microvascular endothelial cells and are easily identified by their elongated elliptical nuclei with a scant surrounding cytoplasm [12]. Their cytoplasm extends to form finger-like projections that traverse the length of the microvessels, enveloping and supporting the vessel wall. Through multiple intercellular junctions and a shared basement membrane, pericytes maintain close physical ties with endothelial cells (Figure 1).

As shown in Figure 1, pericytes encircle the endothelial cells in capillaries and microvessels across both the functional and basalis layers of the endometrium. In contrast, as another type of perivascular cells, adventitial cells mainly reside in the larger vessels within the basalis layer [13]. The adventitial cells in the outermost layer of large vessels contribute to the formation of the vascular structure and stiffness. Functionally, they are involved in stabilizing the blood vessels [14]. Studies utilizing a three-dimensional gel matrix in ex vivo co-culture have shown that the absence of pericytes results in the loss of microvascular integrity [15]. A study conducted on porcine endometrium provided some novel insights into these perivascular cells by revealing the existence of potential mesenchymal stem/progenitor cells situated in the perivascular location.

Additionally, it was suggested that the CD105 and CD146 stromal cells, often more differentiated, could be traced back to their perivascular ancestors [16]. Previous studies have shown controversial results about whether endometrial scratching could improve in vitro fertilization (IVF) outcomes [17]. A recent study suggested that endometrial injury may improve IVF outcomes in women with recurrent implantation failure by influencing perivascular endometrial mesenchymal stem/progenitor cells (eMSCs), which are thought to regenerate the stromal vascular component of the functional layer every month [18].

In the endometrial tissue, it has been found that perivascular cells, including pericytes and adventitial cells, are multipotent cells of mesenchymal origin [13]. Human endometrial pericytes are believed to play a key role in endometrial vascularization, immunomodulation, repair, and reconstruction [19]. However, no consensus exists concerning the source of endometrial pericytes so far. The hierarchical relationship between pericytes and adventitial cells also requires further investigation.

### 2.2. Molecular Markers

Reaching a consensus on how to define pericytes has posed a challenge for several decades. As a highly plastic cell type, their molecular signature, morphological markers, and functional diversity have proven to be both complex and dynamically changing. 

Their complex phenotypic characteristics might be explained by their diverse developmental origins and vessel recruitment patterns, as well as their specific anatomical positions [20]. In organs other than the endometrium, pericyte markers have usually been selected based on a rigorous assessment of their transcriptional abundance, specificity, and uniformity, as informed by single-cell RNA sequencing (scRNA-seq) data [21]. The chosen markers have then been validated through in situ analysis, which has served as an additional selection criterion. Among all of the markers identified so far across multiple organs, there are several universal and stable pericyte markers, such as neural antigen 2 (NG2), platelet-derived growth factor receptor beta (PDGFRb), and regulator of G protein signaling 5 (RGS5) [21]. There is also a consensus in the research community that pericytes typically do not express hematopoietic or endothelial markers like CD45 and CD31 [22]. As of today, pericytes are usually identified by combining information from morphological and molecular markers with the localization of the cell. It is, however, important to interpret the current literature with caution since no definite molecular markers have been identified for endometrial pericytes. Historically, endometrial pericytes have been identified as SUSD2+ cells [23]. Along with the development of sequencing technologies, more molecular markers at the transcriptomic level have been proposed. Recent advancements in scRNA-seq have enabled the identification of a wide range of putative pericyte markers and tissue-specific characteristics. Indeed, our previous scRNA-seq study identified a set of common signature genes for human endometrial pericytes: *RGS5*, Actin Alpha 2, Smooth Muscle (*ACTA2*), Caveolin 1 (*CAV1*), HIG1 Hypoxia-Inducible Domain Family Member 1B (*HIGD1B*), and Collagen Type IV Alpha 1 Chain (*COL4A1*) [24]. Furthermore, the pericyte cluster was divided into two subtypes according to the expression level of *RGS5*: pericyte subtype 1 with high *RGS5* presents as *CSPG4*+, *CNN1*^low^, and *MYH11*^low^, and pericyte subtype 2 shows a *CSPG*-, *CNN1*^high^, *MYH11*^high^ profile [19]. We speculated that pericyte 1 represented a classic mural cell population, while pericyte 2 was more like a smooth muscle or contractile pericyte. In the future, by utilizing genetically encoded lineage markers and lineage tracing technology, it may be possible to track endometrial pericytes as they divide and differentiate them into various cell types, which could aid in characterizing these cells [25]. 

### 2.3. Plasticity

It has been proposed that plasticity is an essential feature of the stem state [26]. Several studies have demonstrated that pericytes exhibit properties akin to MSCs, including multilineage differentiation potential, plasticity, and a similar set of surface markers [13]. The abluminal position seems to be a preferential niche for several types of multipotential progenitor cells, regardless of their tissue of origin. This position enables them to maximize their properties, such as organogenesis, tissue renewal, and regeneration post-injury. 

Pericytes in the endometrium hold high plasticity and the potential for multilineage differentiation. As described above, CD34+ adventitial cells and CD146+ pericytes are two types of perivascular cells derived from the human endometrium that both display MSC phenotypes. It has been reported that CD34+ adventitial cells and CD146+ pericytes can differentiate into endometrial stromal-like cells in vitro [27]. The blood vessel wall harboring these perivascular cell types is considered a source of multipotent cells [22]. One study revealed that endometrial MSCs are multipotent clonogenic pericytes that exhibit self-renewal and lineage-specific pathways, the capacity to adapt to endometrial desquamation and regeneration conditions, and a genetic program indicative of their differentiated lineage, of the stromal fibroblast [28]. In essence, their unique location and potential to differentiate into stromal-like cells grant them the substantial capacity to contribute to endometrial regeneration and repair. 

## 3. The Roles of Pericytes in the Endometrium

### 3.1. Vascularization

Pericytes are critical players in angiogenesis and vascularization, contributing to vasculogenic processes, vessel maturation, capillary permeability regulation, and blood flow control. They support endothelial cells by facilitating extracellular matrix production and providing structural stability to developing blood vessels [28]. Additionally, pericytes produce angiogenic factors such as transforming growth factor beta (TGF-β) and vascular endothelial growth factor (VEGF), which stimulate endothelial cell proliferation and transformation, thereby promoting new vessel formation [29].

In the endometrium, pericytes play several crucial roles, particularly during the late secretory phase and menstruation. As progesterone levels decline, the endometrial vasculature undergoes vascular stasis and vasoconstriction, with a short relaxation of the arterioles resulting in bleeding [30]. During menstruation, hemostasis and vascular repair occur simultaneously, with angiogenesis being essential for endometrial repair. Pericytes also contribute to the maturation of the endometrial vasculature by stabilizing immature basal and proliferating endothelial cells, creating a foundation for further endometrial cell proliferation. Furthermore, pericytes maintain vascular stability and strengthen the endothelial cell wall through direct contact and paracrine regulation [31], secreting and activating signaling molecules such as ANGPT1 and TGF-β [29,31,32]. The proliferative branching length of the endothelial cells during angiogenesis is crucial for establishing a functional capillary network and vascular remodeling. For instance, by generating a diphtheria toxin/*DTR*-mediated pericyte depletion murine model (*DTR*^iPC^ double-transgenic mice), Eilken et al. [33] demonstrated that pericytes regulate endothelial cell proliferation and branching out in retinal angiogenesis through the VEGF-A/VEGFR2 signaling pathway. Thus, we believe that pericytes are indispensable for angiogenesis and vascularization in the endometrium, playing multifaceted roles in supporting the endothelial cells, stabilizing the blood vessels, and facilitating vascular repair and regeneration. 

### 3.2. Immunomodulation

Efficient endometrial remodeling during the breakdown and early proliferative phases requires the involvement of immune responses activated by various immune cells, such as uterine natural killer cells (uNKs), macrophages (uMCs), neutrophils, dendritic cells (DCs), and T cells [34,35]. During the late secretory phase, as progesterone levels decline, the endometrium experiences a localized inflammatory response, resulting in edema and the influx of specific maternal immune cells into the stroma [36]. Numerous molecules released by the pericytes are involved in immunomodulation during the tissue-healing process [37]. In the brain, pericytes are known to participate in immunosurveillance and modulation of neuroinflammation [38]. Similarly, in the endometrium, pericytes play a role in wound healing, which involves massive neutrophil infiltration, immune cell activation, and immunomodulation [39].

Pericytes secrete a basal secretome that includes cytokines and adhesion molecules such as IL-1α, IL-1β, IL-6, E-selectin, and vascular cell adhesion molecule (VCAM)-1, as well as angiogenic factors [40]. The basal secretome of the pericytes plays an important role in tissue-specific inflammation, immunomodulation, tissue repair, regeneration, and preserving vessel integrity. Following exposure stimuli, pericytes act as the frontline for sensing environmental signals and release both an induced secretome unique to the given stimulus and an increased amount of the basal secretome [37,41]. For instance, pericytes in the brain increase the secretion of nerve growth factor (NGF), brain-derived neurotrophic factor (BDNF), and neurotrophin (NT)-3 in response to hypoxia [42].

To our knowledge, studies about the endometrial pericyte secretome are limited. A few studies have shown that endometrial pericytes secrete microvesicles and exosomes containing cytokines that participate in the immune response observed during endometrial shedding and regeneration [43,44]. However, we do not know how endometrial pericytes respond to various pathological conditions. The immunomodulatory functions of pericytes need in-depth investigation in different biological circumstances.

### 3.3. Endometrial Repair and Regeneration

The remarkable regenerative ability of the endometrium indicates the presence of stem/progenitor cells, which are believed to reside in the basalis layer [45]. Purified perivascular cells display a varied mesodermal developmental potential and blend in with conventionally derived mesenchymal stem cells upon in vitro culture [46]. Considering the shared surface makers and functions between MSCs and pericytes, particularly in the endometrium, researchers believe that endometrial pericytes might act as native MSCs in the endometrium [47]. This was also supported by a study where eMSCs in endometriosis had a similar gene expression profile to endometrial stromal fibroblasts in the eutopic endometrium in endometriosis, with the former thought to be the progenitors of the latter [48].

The onset of menstruation is initiated by a decrease in ovarian progesterone levels, resulting in the release of several inflammatory mediators, primarily produced by the cells surrounding the spiral arterioles in the endometrium [9,49]. This hormonal shift may also affect pericytes, which respond to changes in the permeability of the blood vessels and cytokine release. The prominent distribution of progesterone receptors in perivascular cells suggests that progesterone-induced changes in the endometrium are primarily mediated via these cells rather than the endothelial cells themselves [49].

Both in vivo and in vitro studies have implied that pericytes are a potential origin of MSCs, as they can differentiate into various cell types, including endothelial cells, vascular smooth muscle cells, fibroblasts, adipocytes, chondrocytes, and tissue-specific cells [50]. Pericytes respond to changes in the tissue-specific environment by altering their phenotype and also hold stemness properties, making them a potential cell source of endometrial repair and regeneration. For example, one single-cell RNA sequencing study identified a unique population of *PDGFRβ*+ mesenchymal stromal cells that gained a distinct transcriptomic signature specific to the epithelial cells in response to endometrial shedding, suggesting a transition from stromal to epithelial cells [51]. This study provided evidence indicating that stromal fibroblasts may contribute to restoring the luminal epithelium during endometrial regeneration through a mesenchymal-to-epithelial transition. In 2020, another study investigated the regenerative potential of two perivascular stem cell populations in the human endometrium [27]. They found that CD34+ adventitial cells and CD146+ pericytes both showed MSC phenotypes after in vitro culture and could be induced to differentiate into endometrial endothelial-like cells and stromal-like cells. However, after in vivo xenotransplantation and eutopic transplantation into a rat model, these endometrial perivascular stem cells presented limited regenerative potential. Even though there are close similarities between endometrial MSCs and pericytes, no study has investigated the lineage relationship between them so far. In the bone marrow, it has been demonstrated that pericyte progenitors are highly immature cells with greater plasticity and engraftment potential compared to MSCs, indicating that endometrial MSCs might be the progeny of endometrial pericytes [52].

Considering pericytes’ roles in supporting angiogenesis and modulating immune responses and their differentiation potential, their significance in ensuring the endometrium’s overall health and functional performance is indisputable. Future research focused on the mechanisms by which pericytes contribute to endometrial regeneration may shed light on potential therapeutic targets for various uterine disorders.

### 3.4. Endometrial Decidulization

Decidualization denotes the functional and morphological transformation of the endometrial lining and is required for blastocyst implantation [53]. The decidualization process is intricately associated with the unique spatial and temporal expression patterns of several key molecules in endometrial stromal cells. Studies have highlighted the importance of angiotensin II, interleukin-15 (IL-15), and aminopeptidase A in regulating this complex process. Ando et al. [54] documented the premenstrual disappearance of aminopeptidase A from the endometrial stromal cells surrounding the spiral arteries and arterioles, a change that plays a critical role in the preparation of the endometrium for implantation. Moreover, Ahmed et al. [55] localized angiotensin II and its receptor subtypes within the human endometrium, identifying a novel high-affinity angiotensin II binding site that may have implications for vascular regulation during the menstrual cycle. Additionally, Kitaya et al. [56] reported on the expression of IL-15 in the human endometrium and decidua, suggesting its role in immune modulation during early pregnancy.

Several studies indicate that endometrial pericytes also play a crucial role in the process of decidualization. For instance, one study demonstrated that precursors of human decidual stromal cells are pericyte-like cells, expressing pericyte markers and angiogenic factors, and show chemotactic and contractile abilities under the effects of cytokines [16]. Murakami et al. [57] highlighted that the secretome switch during decidualization is a pivotal event that supports the establishment of a receptive endometrial environment, promoting the survival and function of the decidual cells within the niche. An exometabolomic analysis by Harden et al. [58] demonstrated distinct metabolic shifts in decidualizing stromal and perivascular cells, further elucidating the complex interplay between endometrial pericytes and stroma during the decidualization process. Furthermore, research by Gorsek Sparovec et al. [59] provided insights into the fate of SUSD2+ endometrial mesenchymal stem cells, showing that these cells, which include pericytes, undergo specific transformations during decidualization, underscoring their dynamic role in maintaining endometrial integrity and function during early pregnancy.

Collectively, these findings emphasize the dynamic molecular environment within the endometrium and underscore the significance of perivascular stromal cells in the successful decidualization and implantation process. Further knowledge on pericyte function and the molecular mechanisms in the decidualization process may create new insights contributing to our understanding of the establishment and maintenance of early pregnancy.

## 4. Pericytes in Endometrial Pathology

### 4.1. Intrauterine Adhesions and a Thin Endometrium

Intrauterine adhesions, also named Asherman’s syndrome, constitute a gynecological condition that usually results from deep trauma to the endometrium, followed by tissue fibrosis obliterating the uterine cavity partially or completely [60]. Histologically, the endometrial tissue becomes thin, pale, atrophic, and avascular in most cases. Functionally, the endometrium is usually unresponsive to steroid hormones, with poor growth of the glandular epithelium and impaired vascular development, resulting in a thin endometrium [61]. Currently, surgical treatment with resection of fibrotic adhesions is utilized to restore the uterine cavity [62]. It is, however, critical to also restore the function of the endometrium, including regeneration of the parenchymal cells and neovascularization, for patients with fertility requirements.

The fibrosis process following injury is a typical wound-healing process. It is reported that an imbalanced tissue repair response following an acute or chronic injury can lead to fibrosis, characterized by the abnormal accumulation of activated and contractile αSMA+ myofibroblasts [63]. Myofibroblasts release significant amounts of inflammatory mediators, growth factors, and the extracellular matrix (ECM) components, contributing to aberrant ECM remodeling. In recent years, several studies have reported that pericytes might serve as the precursors of myofibroblasts, contributing to organ fibrosis [64,65,66]. It has also been reported that pericytes may be involved in endometrial fibrosis [62]. The pericyte-to-myofibroblast transition is proposed as a key mechanism contributing to various fibrotic conditions [20]. In addition, endometrial MSCs have been regarded as crucial to enabling endometrial remodeling [49].

MSCs occupy a perivascular niche and contain regenerative and immunomodulatory properties. Given these characteristics, MSCs of endometrial and non-endometrial origin (bone marrow, adipose, or placental) have been investigated for therapeutic purposes. However, the specific role of endometrial pericytes in the pathogenesis of intrauterine adhesions warrants further investigation.

### 4.2. Adenomyosis and Endometriosis

Adenomyosis is a prevalent gynecological disorder characterized by the invasion of the endometrial glands into the inner layer of the myometrium, accompanied by muscular infiltration and microenvironmental factors that stimulate the growth of the smooth muscle cells [67]. As a sisterhood disease, endometriosis is characterized by the endometrial glands and stroma residing outside of the uterus [68]. Despite their common occurrence, their etiology remains elusive. Recent studies suggest that the pericytes may play a crucial role in the development and progression of adenomyosis [69]. The multipotency of the pericytes, coupled with their proximity to the myometrial layer, suggests that they may contribute to the pathogenesis of adenomyosis as the origin. A retrospective analysis of histopathological slides revealed the infiltration of the uterine blood vessels in adenomyotic lesions and suggested the presence of mesenchymal-stem-cell-like cells in the perivascular niche [70]. This finding supports the notion that adenomyosis may originate from multipotent vascular pericytes located in the myometrial layer. Furthermore, Sieiński et al. [71] found that adenomyotic interstitial proliferation originates from perivascular interstitial growth, which suggests that the potential pluripotent stem cell population involved in adenomyosis could be pericytes. The potential role of pericytes in the development of adenomyosis is further supported by the observation that adenomyotic lesions exhibit increased vascularity and angiogenesis [72]. Pericytes contribute to vascular remodeling and stabilization, which could facilitate the invasive behavior of the ectopic lesions in adenomyosis. In addition, one study demonstrated that endometrial fibroblasts, as the progeny of eMSCs, exhibited progesterone resistance inherited from the eMSCs and a pro-inflammatory phenotype in endometriosis [48].

Thus, the current evidence suggests that pericytes may play a significant role in the initiation and development of adenomyosis. The function of pericytes in endometriosis needs further exploration. Future studies could focus on understanding the underlying molecular mechanisms by which pericytes may contribute to adenomyosis. Such knowledge is vital to developing novel therapeutic interventions targeting the pericytes for the treatment of adenomyosis and endometriosis.

### 4.3. Abnormal Uterine Bleeding and Related Conditions

Abnormal uterine bleeding (AUB) is a common gynecological condition with heterogeneous etiologies, usually causing a negative impact on women’s quality of life [73]. Idiopathic AUB of endometrial origin is thought to be associated with immature and fragile microvessels [74]. One clinical study demonstrated that low pericyte coverage and rearrangement of the pericytes may play a causal role in idiopathic heavy menstrual bleeding (HMB) and contribute to excessive blood loss [74]. The researchers observed a correlation between reduced pericyte coverage of the endometrial microvessels and the increased expression of VEGF-A, a potent angiogenic factor, suggesting a potential mechanism for abnormal bleeding. In addition to its possible role in HMB, pericytes may also be involved in endometrial calcification. Starostanko et al. suggested that CD34+ adventitial cells could contribute to the pathogenesis of endometrial calcification, which, in turn, may lead to irregular uterine bleeding, chronic pelvic pain, and even infertility [75]. Also, other conditions related to abnormal uterine bleeding, such as polycystic ovary syndrome (PCOS), have been suggested to have altered eMSC gene expression profiles, suggesting underlying endometrial pericytes and possible vasculature abnormalities in women with these conditions [76], which warrants further research [77].

Taken together, the loss of pericytes from the endometrial microvessels may trigger aberrant blood vessel remodeling, resulting in abnormal bleeding patterns. The finding opens up new avenues for pericyte-targeted therapies aiming to restore normal vessel function and maintain homeostasis of the endometrium.

## 5. Future Perspectives

Pericytes are a diverse population of cells that play a crucial role in the maintenance of endometrial homeostasis through vascular remodeling, immunomodulation, repair, and regenerative processes. To date, many critical questions regarding endometrial pericytes remain unanswered, such as their exact localization, characterization of endometrial pericyte sets and subsets, their molecular signatures, the mechanisms responsible for their biological activities, and their involvement in different pathological events. Future research should focus on precisely characterizing their different subtypes, their hierarchical relationships, and their distinct functions both temporally and spatially during endometrial repair and regeneration. Understanding the role of the perivascular microenvironment, including interactions with other cell types and cellular components, will be vital to understanding their functional role in the endometrium. Elucidating the cellular and molecular mechanisms and functions of pericytes will pave the way for more tailored therapies for various uterine disorders. Such advances hold the potential to significantly improve the clinical outcomes for patients by offering more precise and effective treatment options.

## 6. Conclusions

In summary, a deeper understanding of the endometrial pericytes and their role in both healthy cycling endometria and pathological endometria is essential to advancing the field of reproductive medicine. This knowledge will not only enhance our comprehension of endometrial biology but also accelerate the development of innovative therapeutic approaches to managing several uterine disorders.
